# Urban Land Use and Land Cover Classification Using Novel Deep Learning Models Based on High Spatial Resolution Satellite Imagery

**DOI:** 10.3390/s18113717

**Published:** 2018-11-01

**Authors:** Pengbin Zhang, Yinghai Ke, Zhenxin Zhang, Mingli Wang, Peng Li, Shuangyue Zhang

**Affiliations:** 1 Laboratory Cultivation Base of Environment Process and Digital Simulation, Capital Normal University, Beijing 100048, China; zjxczpb@126.com (P.Z.); mingliw@cnu.edu.cn (M.W.); tianming_li@126.com (P.L.); zsycnu@163.com (S.Z.); 2 EarthSTAR Inc., Beijing 100101, China; 3 Beijing Laboratory of Water Resource Security, Capital Normal University, Beijing 100048, China; 4 Beijing Advanced Innovation Center for Imaging Technology, Capital Normal University, Beijing 100048, China

**Keywords:** urban land cover classification, high spatial resolution satellite imagery, deep learning, U-Net, CNN

## Abstract

Urban land cover and land use mapping plays an important role in urban planning and management. In this paper, novel multi-scale deep learning models, namely ASPP-Unet and ResASPP-Unet are proposed for urban land cover classification based on very high resolution (VHR) satellite imagery. The proposed ASPP-Unet model consists of a contracting path which extracts the high-level features, and an expansive path, which up-samples the features to create a high-resolution output. The atrous spatial pyramid pooling (ASPP) technique is utilized in the bottom layer in order to incorporate multi-scale deep features into a discriminative feature. The ResASPP-Unet model further improves the architecture by replacing each layer with residual unit. The models were trained and tested based on WorldView-2 (WV2) and WorldView-3 (WV3) imageries over the city of Beijing. Model parameters including layer depth and the number of initial feature maps (IFMs) as well as the input image bands were evaluated in terms of their impact on the model performances. It is shown that the ResASPP-Unet model with 11 layers and 64 IFMs based on 8-band WV2 imagery produced the highest classification accuracy (87.1% for WV2 imagery and 84.0% for WV3 imagery). The ASPP-Unet model with the same parameter setting produced slightly lower accuracy, with overall accuracy of 85.2% for WV2 imagery and 83.2% for WV3 imagery. Overall, the proposed models outperformed the state-of-the-art models, e.g., U-Net, convolutional neural network (CNN) and Support Vector Machine (SVM) model over both WV2 and WV3 images, and yielded robust and efficient urban land cover classification results.

## 1. Introduction

Urban land use and land cover mapping is a fundamental task in urban planning and management. Very High Resolution (VHR) remote sensing satellite imagery (Ground Sampling Distance (GSD) < 5 m) such as those acquired by QuickBird, IKONOS, GeoEye, WorldView-2/3/4, and GaoFen-2 have shown great advantage in urban land monitoring due to the spatial details they provide. In recent years, many research efforts have been made on urban land use and land cover classification based on VHR imagery [1,2,3,4]. The classification methods can be generally categorized into two classes, i.e., pixel-based methods and object-based methods [2,5,6]. The former defines classes for individual pixels mainly based on the spectral information. In VHR imagery, pixel-based methods may cause salt-and-pepper problems because the spectral responses of individual pixels do not represent the characteristics of the surface object. In order to solve the problem, object-based methods were introduced. In contrast to the pixel-based methods, object-based methods merge neighboring pixels into objects using image segmentation techniques such as Multi-Resolution [7], Mean-Shift [8], or Quadtree-Seg [9] approaches, and the objects are treated as classification units. Object-based classification approaches greatly help reduce the “salt-and-pepper” effects, while image segmentation quality significantly affects the resultant classification accuracies [5,6]. Additionally, analysts need to first arbitrarily select a specific set of object features, or extract representative features using feature engineering techniques as classification inputs. The effectiveness of the selected features also influences classification accuracies. In the complex urban area, the selected features are unlikely to be representative for all land cover types. It is of great need for automatic feature learning from remote sensing imagery instead of using manually selected features.

Artificial intelligence techniques for pattern recognition and computer vision, such as K-means, neural networks (NN), Support Vector Machines (SVM) and Random Forests (RF), have proved to be an effective way for remote sensing image classification. In 2006, deep learning theory was proposed by Hinton et al. [9]. Compared to traditional machine learning techniques such as NN, SVM and RF, deep learning models are highlighted by their emphasis on automatic feature learning from big datasets. They learn to extract discriminative features within the modeling process; thus, they do not require prior feature design processes. In computer vision communities, deep learning, particularly Convolutional Neural Networks (CNNs), have been successfully applied for image categorization, target detection and scene understanding [10,11,12,13,14]. Generally, typical CNNs include convolutional layers and pooling layers, activation function introducing nonlinearity, followed by fully connected layers as classifiers. The first application of CNN in remote sensing was the extraction of road networks and buildings by Mnih and Hinton [15] in 2010. Recently, CNNs have been used for per-pixel semantic labeling (or image classification) of high resolution remote sensing image. Mnih and Hinton [15] proposed a CNN architecture for aerial image labeling. Wang et al. [16] applied a three-layer CNN structure and Finite State Machine for road network extraction. Hu et al. [17] used a pre-trained CNN to classify different scenes in high resolution remote sensing imagery. Längkvist et al. [18] applied pixel-based CNN to classify 0.5 m true color aerial imagery into five land cover classes including vegetation, ground, road, building and water with the aid of a digital surface model (DSM). Maltezos [19] utilized CNN to extract buildings from ortho-imagery with the aid of height information. Pan and Zhao [20] proposed a central-point enhanced CNN model for land cover classification based on GaoFen-2 4-band satellite imagery over rural areas. Zhang et al. [21] combined multi-layer perception model and CNN to classify very fine spatial resolution aerial imagery in both urban and rural area.

In 2015, a fully convolutional neural network (FCN) was proposed by Long [22]. FCN replaces the fully connected layers in CNN with up-convolutional layers and concatenates with a shallow, finer layer to produce end-to-end label. As the standard CNN operates in an “image-label” manner, the “end-to-end” labeling mode in FCN is more suitable for pixel-based image classification, i.e., labeling each pixel to a certain class. The framework of FCN has also shown great potential in remote sensing image classification. Sherrah [23] proposed a no-downsampling FCN framework for semantic labeling over International Society for Photogrammetry and Remote Sensing (ISPRS) Vaihingen and Potsdam benchmark datasets, which are public aerial false-color imagery with 9 cm spatial resolution and accompanied with DSM derived by Light Detection and Ranging (LiDAR) measurements. Based on the same datasets, Audebert et al. [24] implemented a multi-scale FCN. Sun et al. [25] proposed an ensemble FCN model. Maggiori et al. [26,27,28] compared CNN and FCN models for aerial image labeling, and presented a multilayer perceptron (MLP) framework based on FCN model for large-scale aerial image classification. Fu et al. [29] employed FCN and conditional random fields (CRF)-based post-processing for land cover classification over GF-2 and IKONOS imagery. Our literature review indicates that most of the existing studies, except for Fu et al. [24], were conducted based on the ISPRS Vaihingen and Potsdam benchmark datasets. Although these public datasets provide imagery and reference data sources for more efficient modeling and model comparisons, operational land cover and land use classification in large urban areas often rely on satellite imagery with relatively coarser spatial resolution (e.g., 0.5 m), and accurate DSMs are not always available. It is also not clear how spectral bands affect deep-learning-based image classification accuracy.

U-Net model proposed by Ronneberger et al. [30] is an improved FCN model characterized by symmetrical U-shaped architecture consisting of symmetric contracting path and expansive path. It combines low level features with detailed spatial information with high level features with semantic information to improve segmentation accuracy, and has attained promising results in one-class segmentation tasks such as biomedical image segmentation [30], road network extraction from aerial imagery [31,32], building extraction [33], and sea-land segmentation from Google earth images [34]. For multi-class labeling tasks such as land cover classification, contextual information at multiple scales are important because features of different land cover types or ground objects usually are presented at various scales. However, this information is not incorporated in the original form of U-Net model.

Convolution is an essential step in both CNN and FCN models as it allows for models to increasingly learn abstract feature representations. However, the pooling operations and convolution striding between layers in convolutional process reduce the spatial resolution of the feature map, thus leading to the loss in spatial details. On the other hand, ground objects in remote sensing imagery usually exist in multiple scales. In order to learn the contextual information at multiple scales, Chen et al. [35,36] proposed the Atrous Spatial Pyramid Pooling (ASPP) technique, which employs multiple parallel atrous convolution filters with different rates. With atrous filters increasing the effective field-of-view in convolution, the spatial pyramids allow features to be captured at multiple scales. The multi-scale feature capturing capability of ASPP is especially useful in land cover and land use classification task in the complex urban area which is characterized by high spatial heterogeneity.

Inspired by ASPP and U-Net model, we proposed a framework that takes advantage of strengths from both ASPP and U-Net, called the ASPP-Unet model. The proposed model is built based on the U-Net architecture. The difference between our ASPP-Unet and U-Net is that contextual information is incorporated through ASPP strategy in the bottom layer of the network. In addition, we further incorporated the idea of deep residual learning [37] to our ASPP-Unet model, called ResASPP-Unet model. In this study, we trained and tested our models on WordView-2 and WorldView-3 imageries covering a large urban area in Beijing. The impact of spectral bands, the number of model layers and initial feature maps on the classification accuracy were investigated. In addition, we compared our approaches with U-Net, CNN and SVM approaches. Detailed description of the methods and results are shown in Section 2 and Section 3. Section 4 discusses the results, and Section 5 draws the conclusions.

## 2. Methods

In this study the ASPP-Unet and ResASPP-Unet models for urban land cover classification from VHR imagery are proposed. Like other supervised classification methods, the classification procedure consists of training stage and test stage. Unlike conventional training strategies that use randomly-selected pixels or image objects as training samples, the training samples in our model are pairs of image patch and its corresponding ground truth patch with each pixel labeled with a certain class. Through back-propagation and iterations, the optimal sets of parameters are learned. In the classification stage, the trained ASPP-Unet and ResASPP-Unet models are then performed on an input image to predict the class for each pixel. The details of the network architecture are described in Section 2.1. The network training stage and classification stage are presented in Section 2.2 and Section 2.3, respectively.

### 2.1. Network Architecture

#### 2.1.1. ASPP for Multi-Scale Feature Extraction

**Figure 1:**
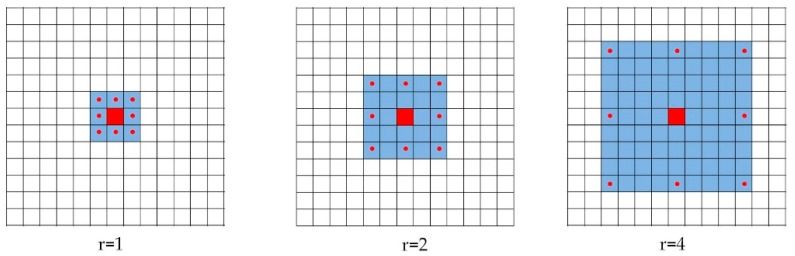
Atrous convolution over 2D imagery with rate = 1, 2 and 4. The red pixel denotes the target pixel and the red-dotted pixels denote the pixels involved in convolution.

Inspired by Chen et al. [35], we designed the atrous convolution for dense feature extraction with variant field-of-view. In the image processing case, the atrous convolution can be expressed by:

(1) $$y\left[i,j\right]=\sum_{k=1}^{K}x\left[i+r\cdot k,\;j+r\cdot k\right]w\left[k\right],$$

where y[i,j] is the atrous convolution output, x[i,j] is the input signal, w denotes the convolution kernel (filter) with length *k*, and *r* denotes the rate parameter corresponding to the stride with which the input signal is sampled. When rate *r* = 1, the atrous convolution is actually the standard convolution. Figure 1 illustrates the atrous convolution on an imagery with 3 × 3 kernel and rate *r* = 1, 2 and 4 for the target pixel. It can be seen that higher sampling rates indicate larger field-of-view (FOV). Compared to the standard convolution filter that requires more parameters to enlarge FOV, the atrous filter enlarges FOV without increasing the number of parameters to be calculated, thus saves the computation resources. ASPP follows this idea and implements multiple atrous convolutional layers with different sampling rates in a parallel manner. The multi-scale features are then fused to generate a final feature map [36].

#### 2.1.2. ASPP-Unet Architecture

U-Net model was first proposed by Ronneberger et al. [30]. The original form of U-Net architecture consists of a contracting path and a mirrored expansive path. The contracting path extracts high level features by convolution and pooling operations, while the spatial resolution of the feature maps reduces. The expansive path attempts to restore the resolution of feature maps using up-convolution operations. For each level of the contracting path, the feature maps are passed over to the analogous level in the expanding path, allowing for contextual information propagating through the network. 

**Figure 2:**
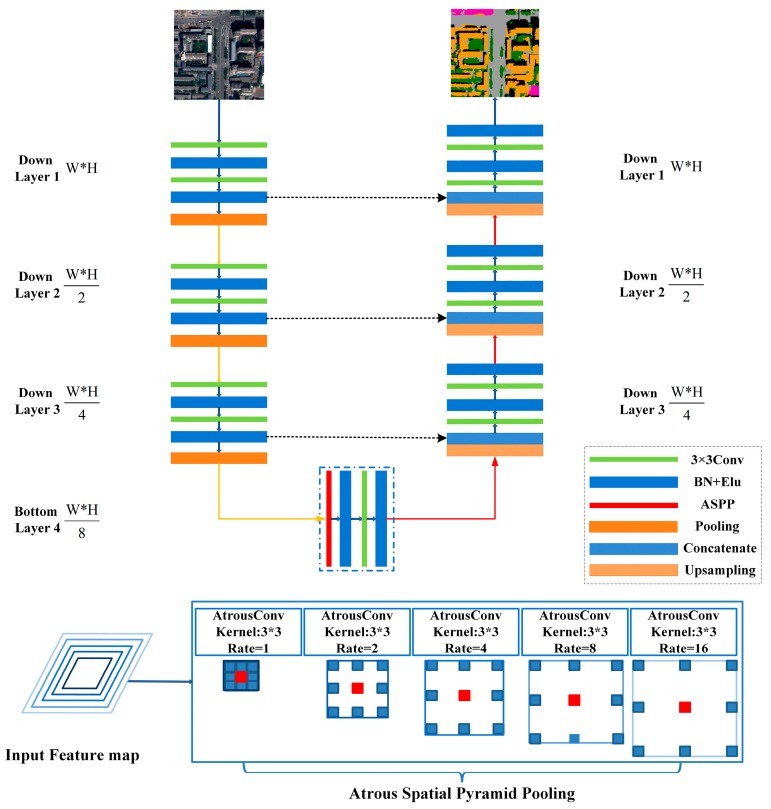
Atrous spatial pyramid pooling (ASPP)-Unet architecture. This example has 7 layers.

Based on the U-Net model, we proposed a novel ASPP-Unet model by incorporating ASPP approach [30]. Figure 2 illustrates the ASPP-Unet model architecture. Similar as U-Net, the contracting path follows a classical CNN model. Each down layer contains two sequential unpadded 3 × 3 convolutions. Each convolution operation is followed by an element-wise activation function. The number of feature maps is doubled after the previous convolution. In this study, we use exponential linear unit ((Elu) [38] as primary activation function (Equation (2)):

(2) $$f\left(x\right)=\{\begin{array}{cc} x & \left(x>0\right)\\ \alpha \left(\exp\left(x\right)-1\right) & \left(x<0\right) \end{array},$$

where *α* is the hyper-parameter between 0 and 1. Compared to rectified linear unit (ReLu) activation function, Elu can alleviate the vanishing gradient problem via the identity for positive values [38] and also accelerate learning in deep neural networks so as to alleviate the computation complexity. Before each Elu activation function, we implemented batch normalization. Batch normalization normalizes the inputs to the layers, usually a mini-batch, by both mean and variance. It remedies internal covariate shift, and thus speeds up training process [39]. Between the upper and lower layers in the contracting path, a 2 × 2 max pooling operation is applied for down sampling, leading to reduction in the resolution of feature maps. Figure 2 shows that the size of feature maps at the bottom layer of contracting path reduces to one-eighth of the original image (*W*/8 × *H*/8 vs. *W* × *H*, where *W* and *H* denote width and height of an image patch, respectively). The bottom layer of the standard U-Net model follows the same structure of the down layer, i.e., two sequential convolution, batch normalization and Elu operations. In the proposed ASPP-Unet model, we exploited ASPP approach in the bottom layer. As shown in Figure 2, five 3 × 3 atrous convolutions with rates *r* = 1, 2, 4, 8, and 16 are implemented in a parallel way to the input feature maps of bottom layer, and then fused together by sum operation. After that, standard convolution and Elu are performed. 

The expansive part has the same number of up layers as the down layers. A 2 × 2 up-convolution operation is first employed from the bottom layer of contracting path. The up-convolution is conducted with transpose convolution operation, which is the inverse process of convolution. The transpose convolution halves the number of feature channels and increases the resolution to be equal to the corresponding down layer. The feature maps are then concatenated with the mirrored feature maps in the contracting path through skip connection. In order to conduct the concatenation, the feature maps at the contracting path are cropped according to the size of the current up layer feature maps. The concatenation operation allows for the high resolution features from contracting path to be combined with the coarse resolution features resulted from the upsampling procedure, so that the contextual information can be propagated through the network. Then, two sequential 3 × 3 convolutions with each followed by batch normalization and Elu activation function are applied on the concatenated feature map. As illustrated in Figure 2, the feature map of the analogous level of the down layers and up layers have the same size. The spatial resolution of the feature map in the last up layer is same as that of the input image. Finally, a 1 × 1 convolution is applied to map the features from the last up layer to the number of classes, and Softmax layer is applied to transform the features to the probability belonging to each land cover type. Through these procedures, ASPP-Unet retains a large number of feature maps in contracting path and extends it by a symmetric U-shaped pattern; in addition, it captures multi-scale deep features by employing multiple parallel filters with different rates.

#### 2.1.3. ResASPP-Unet Architecture

**Figure 3:**
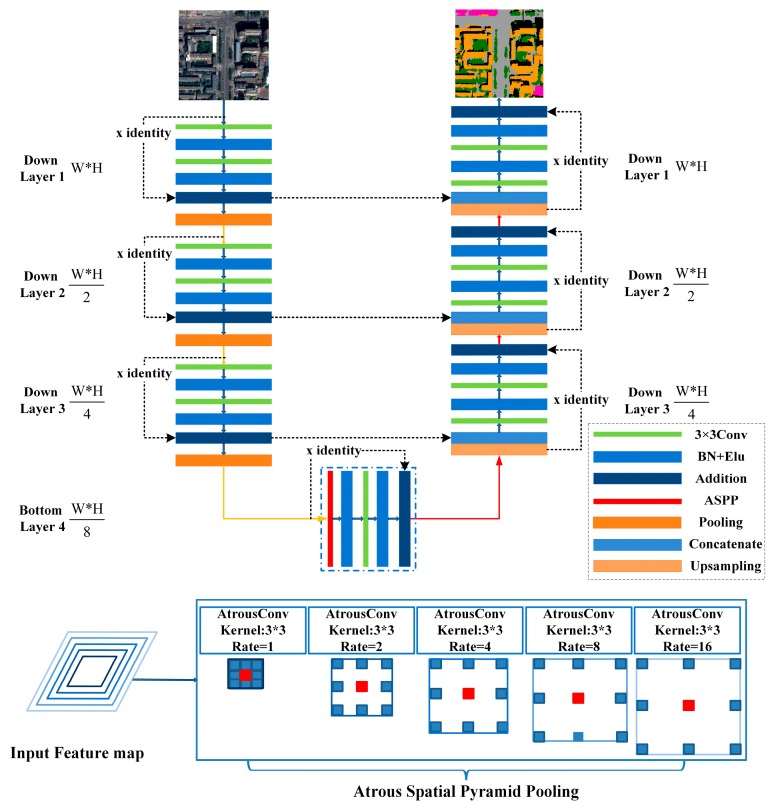
ResASPP-Unet architecture. This example has 7 layers.

As shown in Figure 2, each layer in the contracting path of the ASPP-Unet model mainly relies on convolution operations to learn high-level features. During recent years, research has reported that classification accuracy does not necessarily increase with the increase of convolutional layers, especially for small number of training samples [40]. The deep residual learning proposed by He in 2016 [37] addressed this issue by adding shortcut connections between every few stacked layers to build residual blocks. We further modified our ASPP-Unet model by adding identity mapping from the input of each layer to the output of the same layer. Thus, each layer forms a residual block illustrated as:

(3) $$\begin{array}{c} y_{l}=h\left(x_{l}\right)+F\left(x_{l},\left\{W_{i}\right\}\right)\\ x_{l+1}=f\left(y_{l}\right) \end{array}$$

where $x_{l}$ and $x_{l+1}$ are the input and output of the *l*-th residual block, $F\left(x_{l},\left\{W_{i}\right\}\right)$ represents the residual mapping to be learned, $f\left(y_{l}\right)$ is an activation function and $h\left(x_{l}\right)$ is the identity mapping function, with a typical form of $h\left(x_{l}\right)=x_{l}$. In this study, we employed residual blocks in both contracting path and expansive path as illustrated in Figure 3. Within each block, the layer structure remains the same as ASPP-Unet except for the shortcut connection part, which performs an identity mapping from the shallower layer and then addition to the current layer. The new architecture is named ResASPP-Unet model.

### 2.2. Datasets and Network Training

**Figure 4:**
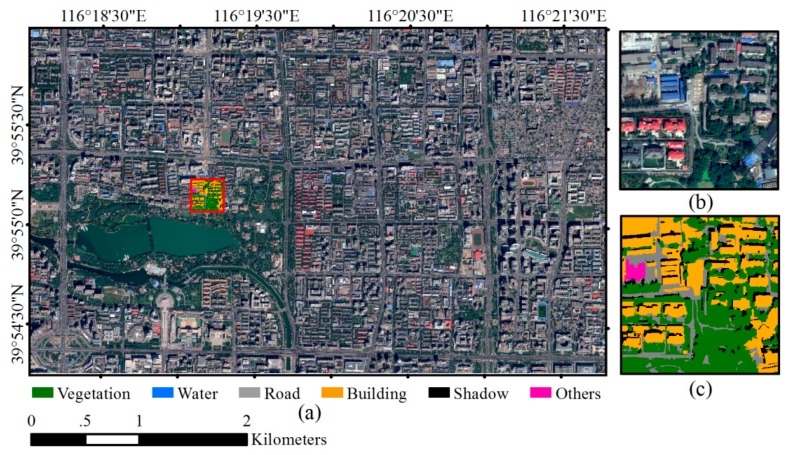
WorldView-2 (WV-2) imagery and an example of reference data. (**a**) WV-2 multispectral imagery; (**b**) an example of sampled image patch; (**c**) ground truth of the image patch.

**Figure 5:**
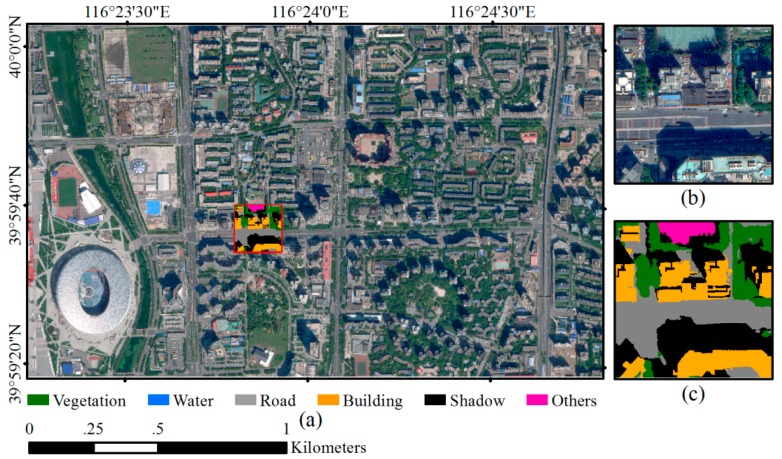
WorldView-3 (WV-3) image and an example of reference data. (**a**) WV-3 multispectral imagery; (**b**) an example of sampled image patch; (**c**) ground truth of the image patch example.

The cloudless WV2 satellite imagery acquired on 14 September 2012 and the WV3 satellite imagery acquired on 3 September 2014 over Beijing urban areas were used in this study (Figure 4 and Figure 5). WV2 imagery covers part of Haidian and Xicheng Districts with area of 17.22 km^2^, and WV3 imagery covers part of Chaoyang District with area of 3.06 km^2^. The WV2 image contains one panchromatic band (450–800 nm) with 0.5 m GSD and 8 multispectral bands with 2 m GSD including coastal (400–450 nm), blue (450–510 nm), green (510–580 nm), yellow (585–625 nm), red (630–690 nm), red edge (705–745 nm), Near Infrared 1 (NIR1) (770–895 nm) and NIR2 (860–1040 nm). The WV3 image has the same eight multispectral bands with 1.2 m GSD and one panchromatic band with 0.3 m GSD. For both WV2 and WV3 imageries, panchromatic image is fused with multispectral image using the nearest-neighbor diffusion-based pan-sharpening algorithm (NNdiffuse) [41] to produce 0.5 m 8-band WV2 imagery and 0.3 m 8-band WV3 image. Both images are projected into UTM WGS84 50N geo-referenced coordinate system, and all the spectral channels are normalized with z-score method [42].

Both study sites covered by WV2 and WV3 imagery contain typical urban land cover types and represent Chinese urban landscape where high-rise buildings interlace with low-rise buildings, and new buildings and historic buildings coexist. Furthermore, the spectral and geometric characteristics of building roofs are diverse due to different roof materials and building types, which may lead to confusions with other surface land cover. Dominant land covers include vegetation, water, road and buildings. Open spaces such as school playground and construction site are also scattered in the study areas.

In order to train and validate the models, a total of 55 WV2 image patches and 3 WV3 image patches with the size of 588 × 588 pixels are cropped from the pan-sharpened WV2 and WV3 image, respectively. All image patches are fully annotated as ground truth (Figure 4c and Figure 5c). Six land cover classes including “vegetation”, “building”, “road”, “water”, “shadow” and “others” are identified for each pixel of the image patch by visual interpretation assisted with automated image segmentation conducted in eCognition software [43]. Each segmented object is visually examined and edited carefully to ensure that the boundary of the object is correct. Among the six land cover types, “others” represents open spaces that accounts for only around 1.4% of the whole study area. In this study, 50 WV2 image patches with their ground reference pairs were randomly selected and used as training samples to build both ASPP-Unet and U-Net classification models. The remaining 5 WV2 image patches and 3 WV3 image patches were used to test the performance of the classification.

The general training procedure is illustrated as follows. First, the training image-ground truth pairs are input to the network, and the weights of the network are initialized. Second, the Softmax function is applied on the last feature map generated from the network to predict the dense distribution of the classes. Then the cross entropy loss is calculated based on the training samples and initial parameters. The loss is then back-propagated through the network and the network parameters are updated. 

In this study, we modified the input layer to be suitable for 3, 4 or 8 image channels. The weights of the network were initialized using a truncated normal distribution recommended by Collobert et al. [44]. The Softmax function (Equation (4)) calculates the probabilities of each class over all six classes for a given pixel.

(4) $$p\left(z_{j}^{i}\right)=\frac{\exp\left(z_{j}^{i}\right)}{\sum_{k=1}^{K}\exp\left(z_{k}^{i}\right)},$$

where $(z_{1}^{i}$, $z_{2}^{i}$, …, $z_{K}^{i})$ denotes the prediction vector for a given pixel *i*, *K* is the number of classes (*K* = 6 in our dataset), and $p\left(z_{j}^{i}\right)$ is the probability of the pixel *i* belonging to the class *j*. As the original form of U-Net was designed to deal with one-class identification problem such as cell extraction in medical image or road extraction [30,31,32,33,34], here we modify the cross entropy loss function for multi-class classification (Equation (5)):

(5) $$loss=\frac{1}{N}\overset{N}{\underset{i=1}{\sum }}\overset{K}{\underset{j=1}{\sum }}y_{j}^{i}\log\left(p\left(z_{j}^{i}\right)\right),$$

where *N* is the number of pixels in the input image, $y_{j}^{i}$ denotes the ground truth label of the pixel *i* which is represented with one-hot vector. The network parameters are updated using momentum-based Stochastic Gradient Decent (SGD) and mini-batch strategy [45]. In this study, the initial learning rate value was 0.1 and decay rate was 0.95 at every train epoch with the momentum of 0.2. The networks were trained with 100 epochs and every epoch contained 10 iterations until no further improvement on the accuracy is achieved. To avoid the overfitting problem, dropout strategy [46] was utilized with a probability of 0.75 and the sparse constraint *L*_2_ regularization was added. 

### 2.3. Classification Using the Trained Network

The trained ASPP-Unet and ResASPP-Unet models can be directly used to predict the whole WV2 and WV3 imageries regardless the size of the image. However, due to the limitation of the memory, in this study we cropped the WV2 and WV3 images into image tiles with 1024 × 1024-pixel size using a sliding window approach to ensure that each pair of neighboring tiles have 188 overlapping pixels around the borders. The prediction of each image tile resulted in 836 × 836 labeled image, and then all labeled image tiles were mosaicked to produce the whole labeled image.

## 3. Experiments and Comparisons

### 3.1. Experiments

For the task of semantic labeling in remote sensing community, a majority of the existing studies implement deep learning networks on three-band true color [31,32,34] or color-infrared (CIR) images [25,26,27,28,29] as input, as most deep learning networks developed by computer vision community mainly deal with RGB images. As WV2 and WV3 offer 8-band spectral information, it is necessary to evaluate the effect of more spectral bands on the classification accuracy. In this study, the aforementioned 8-band image samples were used to generate 4-band image samples composed of R, G, B and NIR band layers, true color image samples composed of R, G and B band layers (RGB) and color-infrared images composed of R and G and NIR band layers (CIR). The ResASPP-Unet, ASPP-Unet and U-Net models were trained and tested with each of the four sets of image patch samples. 

Layer depth is an important parameter in deep learning networks. It is widely believed that the advantage of deep learning mainly comes from the abstract hierarchical feature extraction through the deep hidden layers. Model generalization capability may degrade with insufficient layer depth while the backward gradient may vanish as the layer gets deeper. The number of initial feature maps (IFMs) is another key parameter as the feature maps determine the representation capacity of hidden layers. IFM refers to the feature maps between the input image and the first hidden layer. In this study, the ResASPP-Unet, ASPP-Unet and U-Net models with layer depths of 5, 7, 9, 11, 13 and the number of IFMs of 32, 48, 64 and 80 were implemented in order to evaluate the effects of the network parameters on model performances. As a result, a total of 80 models (i.e., 5 layer depths and 4 IFMs with 4 sets of training samples) for each of the ResASPP-Unet, ASPP-Unet and U-Net were trained and tested. Table 1 summarizes the parameters used to train the models. All models were implemented in Tensorflow framework.

### 3.2. Comparison Setup

The ResASPP-Unet, ASPP-Unet and U-Net models with the parameters that produced the highest accuracy were compared with the following CNN [47] and SVM models [48]. All models were evaluated using overall accuracy (OA), kappa coefficient (*k*), user’s accuracy (UA), producer’s accuracy (PA) and *F*_1_ score (*F*_1_) which is represented as the harmonic mean of UA and PA (Equation (6)).

(6) $$F_{1}^{i}=2\times \frac{{\mathrm{UA}}_{i}\times {\mathrm{PA}}_{i}}{{\mathrm{UA}}_{i}+{\mathrm{PA}}_{i}}$$

#### 3.2.1. CNN

**Figure 6:**
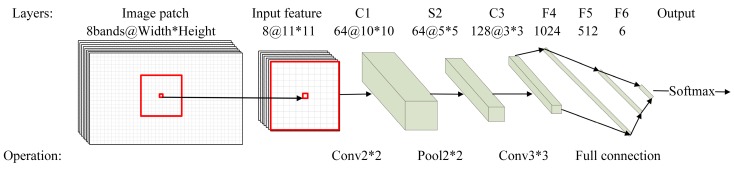
Convolutional neural network (CNN) model architecture.

As patch-based CNNs produce excessive reduction of the resolution and border discontinuity artifacts in the classification map [26,29], in our study a pixel-based CNN was constructed (Figure 6) following [47]. As illustrated in Figure 6, the network is composed of a successive convolution, max-pooling and fully connected layers. The input of the network is the cubic window with *n* × *n* × *b* neighborhood pixels around each central pixel, where *n* and *b* refer to the size of the square window around the center pixel and the number of input channels, respectively. The first convolution layer performs 2 × 2 convolution with Elu activation function and results in feature maps with 10 × 10 size with 64 channels. The max-pooling layer is then followed and produces 5 × 5 feature maps with 64 channels. After the max-pooling operation, the second convolution layer performs 3 × 3 convolution and produces 3 × 3 feature maps with 128 channels. In the final stage, three fully connected layers are used to map the high level features extracted by the convolutional-max-pooling layers to a 1D feature vector with one neuron per class (6 classes in the study). The Softmax function is then applied to obtain the probability distribution of the six classes of land cover. The dropout rate of 0.75 was applied to alleviate overfitting problem. 

In order to train and validate the CNN model, a total of 104,419 pixels are extracted from the 50 training image patches using stratified random sampling strategy. The window size and number of input channels were set as *n* = 11 and *b* = 8, i.e., for each pixel, pixel values of all 8 bands within the surrounding 11 × 11 window were extracted as inputs. Suitable window size should cover sufficient ground area so that edges and textures of objects can be discerned. We tested different window sizes with 7, 9, 11, 15 width and height, and found that 11 × 11 window produced the best accuracy. This is consistent with [49], which also found that CNN with window covering ground area with 5 m × 5 m performs the best. Similar as ASPP-Unet and U-Net implementation, the entropy loss value was then calculated based on the input training samples and initial model parameters, and then back-propagated through the network and updated the model parameters with SGD with momentum. 

#### 3.2.2. SVM

The SVM proposed by Cortes and Vapnik [48] is a kernel-based supervised classification algorithm. The basic concept is that the algorithm attempts to find the optimal hyper plane that maximizes the margin between classes using a kernel function. SVM has been widely used for remote sensing image classification tasks [50,51,52] and has proven to be a powerful machine learning algorithm. To be consistent with CNN, we used the 104,419 pixels as training sample with the 8-band spectral values as input features. The model training and test were implemented in LibSVM software [53]. Radial Basis Function kernel (RBF) was used as the kernel function. In SVM, the penalty factor *c* controls the trade-off between training error and model complexity. A small *c* value may increase the training error while a large *c* value may increase model complexity and lead to the overfitting problem. RBF kernel width *γ* represents the influence radius of samples selected as support vectors. Large value of *γ* may cause high bias and low variance of models, and vice-versa. In this study, optimal *c* and *γ* were determined using grid search and cross-validation strategy. Based on the grid search, the optimal parameters of the model were *c* = 2.0 and γ = 8.0. 

### 3.3. Results

#### 3.3.1. Performances of Res-ASPP-Unet, ASPP-Unet and U-Net

**Figure 7:**
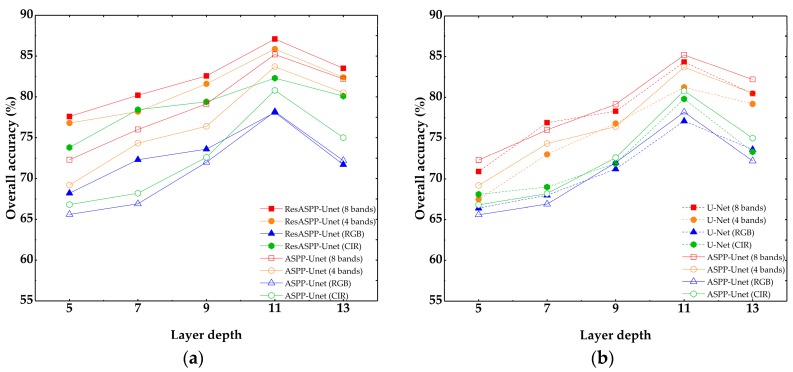
Overall accuracies of ResASPP-Unet, ASPP-Unet and U-Net models with 64 initial feature maps (IFMs). (**a**) ResASPP-Unet and ASPP-Unet; (**b**) ASPP-Unet and U-Net.

Figure 7 illustrates the overall accuracies of ResASPP-Unet, ASPP-Unet and U-Net models with layer depth ranging from 5 to 13, and all models are trained using the four sets of input training samples with different combinations of spectral bands. All models have 64 IFMs. The overall validated accuracies were calculated based on the 5 test WV2 image patches. Figure 7 shows that the test accuracies of all three models initially rise up with increasing layer depth for a given training dataset. For example, when 8-band image patches were used as input, the overall accuracy of the 5-layer ASPP-Unet model is 72.3%; the accuracy increases rapidly with the augment of layer depth and reaches 85.2% when layer depth increases to 11; using the same input datasets, the 5-layer U-Net model produces accuracy of 71.6%, and the 11-layer model produced accuracy of 84.4%. In contrast, the ResASPP-Unet model does not vary as much as the ASPP-Unet and U-Net when the networks get deeper. The accuracy of the ResASPP-Unet with 8-band imagery as input increases from 77.6% to 87.1%, and that of the model with 4-band imagery as input increases from 76.8% to 85.8% as layer depth increases from 5 to 11. For all models, however, networks with deeper layers do not always perform better. Regardless of the input image used, the overall accuracy of each of the three models is the highest when the layer depth is 11, and it drops slightly when the layer depth increases to 13. All three perform the best when 8-band image samples were used as input training datasets. The rank of the overall accuracies model is 8 bands > 4 bands > CIR > RGB. When the layer depth is 11 in ResASPP-Unet model, the overall accuracies are 87.1%, 85.8%, 82.3% and 78.1% for 8-band images, 4-band images, CIR images and RGB images, respectively. And for the ASPP-Unet model, the overall accuracies are 85.2%, 83.2%, 80.3% and 77.6%, respectively. With the same layer depth and input datasets, the ASPP-Unet performs slightly better than U-Net in most cases, and the ResASPP-Unet performs the best among the three models. The highest accuracy was obtained by 11-layer ResASPP-Unet model trained with 8-band image patches (87.1%, “ResASPP-Unet (8 bands)” in Figure 7), which is higher than that of ASPP-Unet model (85.2%, “ASPP-Unet (8 bands)”) and U-Net model (84.7%, “U-Net (8 bands)”), although they are trained using the same set of image patches.

**Figure 8:**
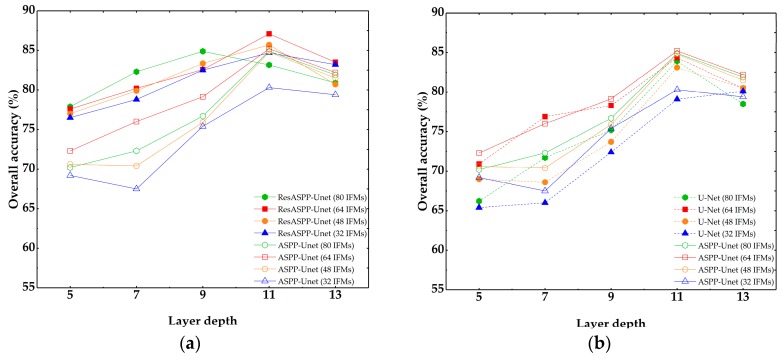
Overall accuracies of ResASPP-Unet, ASPP-Unet and U-Net models with 8-band image samples as input. (**a**) ResASPP-Unet and ASPP-Unet; (**b**) ASPP-Unet and U-Net.

Figure 8 shows the overall accuracies of ResASPP-Unet, ASPP-Unet and U-Net models with the number of IFMs ranging from 32 to 80 and layer depth ranging from 5 to 13. All models are trained using the 8-band image patch samples. It can be seen that, regardless of the IFMs used, the accuracy of the networks increased as the model goes deeper. The accuracy reaches the maximum when the layer depth is equal to 11, and then decreases slightly when the layer depth increases to 13, except for the ResASPP-Unet model with 80 IFMs. The models with 32 IFMs produce considerably lower accuracies than the models with 48, 64 and 80 IFMs. Almost all models yield improved accuracies with the increase of IFM number, and reach the highest accuracy with 64 IFMs. With 80 IFMs, the model performances decreased slightly. For the ResASPP-Unet model, the overall accuracies are 84.7%, 85.7%, and 87.4% when the layer depth is 11 and the number of IFMs is 48, 64 and 80, respectively. For the ASPP-Unet model, the overall accuracies are 83.9%, 85.2% and 85.0%, respectively. The overall accuracies of 11-layer U-Net model are 83.1%, 84.3% and 83.9% with IFMs of 48, 64 and 80, respectively, which are lower than those of ASPP-Unet and ResASPP-Unet models.

**Figure 9:**
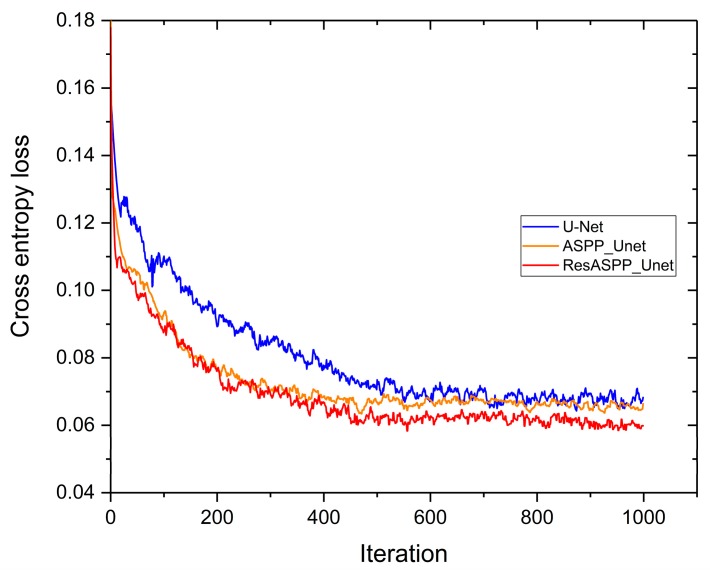
The training entropy loss of U-Net, ASPP-Unet, ResASPP-Unet for WV2 images. The three models have been executed using 100 epochs with 10 iterations per epoch.

Figure 9 demonstrates the cross entropy loss tendency of the training process of ResASPP-Unet, ASPP-Unet and U-Net models with 11 layers and 64 IFMs. After 1000 iterations, all three models have been sufficiently trained and acquired relatively low loss. Among them, the ResASPP-Unet got the minimum training error (nearly 0.06). Compared to U-Net, the ASPP-Unet and ResASPP-Unet converged faster at the early stage of model training. In addition, when the ASPP-Unet model nearly approached the lowest loss, the ResASPP-Unet still has the potential to optimize.

#### 3.3.2. Comparisons with CNN and SVM Models

The ResASPP-Unet, ASPP-Unet and U-Net models with a layer depth of 11 and 64 IFMs were compared to the CNN and SVM models. All models were trained by the WV2 8-band training samples and applied to predict pixel class labels for both WV2 and WV3 test images. Table 2 lists the test accuracies over WV2 image as well as the training accuracies. It can be seen that ResASPP-Unet model achieves the best performance compared to other models. The overall accuracy is 87.1% and the Kappa coefficient is 0.820. ASPP-UNet model obtains slightly lower performance with 85.2% overall accuracy and 0.793 kappa coefficient. SVM produces the lowest accuracy whose overall accuracy is only 71.4% and kappa coefficient is lower than 0.65. For all models, vegetation is the most accurately distinguished class, with *F*_1_ values higher than 0.91. For SVM model, the UA of class water is only 55.9%, indicating that nearly half of the “water” pixels were wrongly classified. In contrast, the deep learning models, namely CNN, U-Net, ASPP-Unet and ResASPP-Unet yielded UA of “water” higher than 80%. In addition, these deep learning models also produce considerably higher accuracies for “road” and “building” classes compared to SVM, which are easily confused in urban land cover classification tasks. Especially, the UA values of ResASPP-Unet for most of the 6 categories are the highest among the four deep learning models. Neither U-Net nor ASPP-Unet detects “other” class. Interestingly, CNN model achieved the highest training accuracy among the four models, which may be attributed to the overfitting. The training accuracies of the SVM and CNN models are much higher than the test accuracies, while the U-Net-based models produced approximate training and test accuracies. 

**Figure 10:**
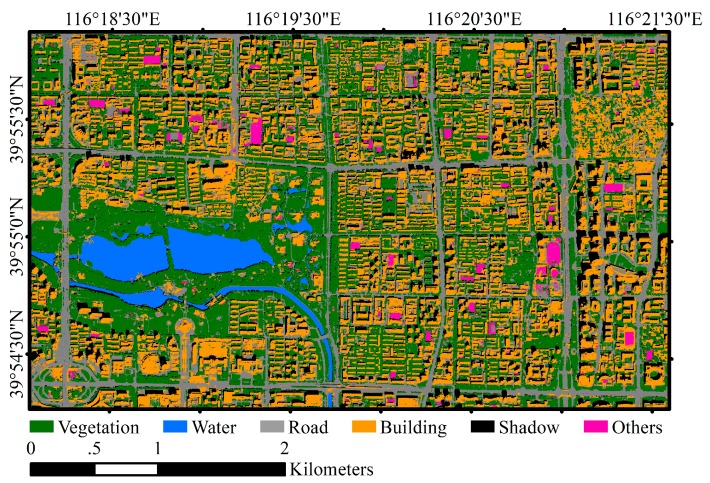
Urban land cover classification map over the WV2 imagery using ResASPP-Unet model.

**Figure 11:**
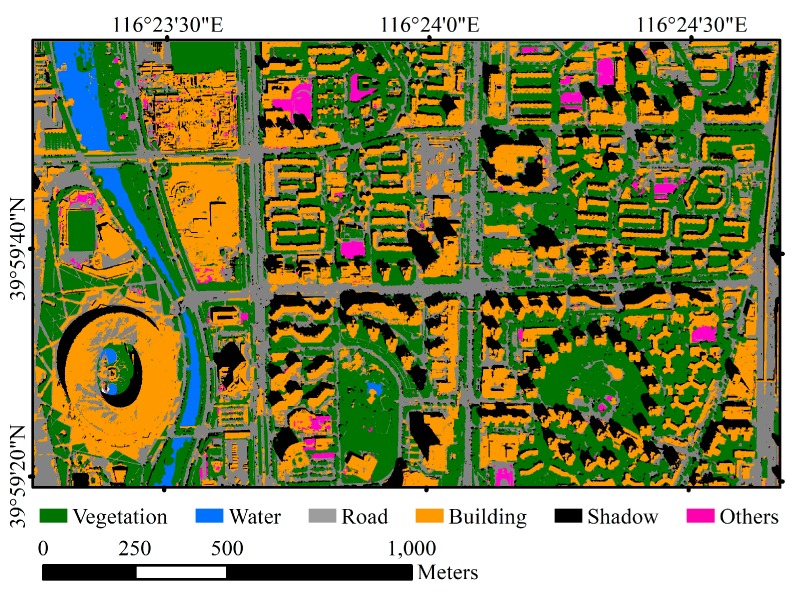
Urban land cover classification map over the WV3 imagery using ResASPP-Unet model.

When the four models trained with WV2 images were applied to predict WV3 images, the performances of all models decrease, among which the SVM produces the greatest degradation in the accuracy (OA = 51.3%) (Table 3). In contrast, CNN, U-Net, ASPP-Unet and ResASPP-Unet generate slight decrease in the OA values (79.2%, 81.2%, 83.2% and 84.0%, respectively). Regardless of the images used, ResASPP-Unet achieved the best performance compared to other models (OA = 84.0%, kappa = 0.787), and ASPP-Unet follows (OA = 83.2%, kappa = 0.774). The WV2 and WV3 image classification results using the ResASPP-Unet models are shown in Figure 10 and Figure 11, which visually illustrates that most of the pixels are labeled correctly, and also verify the ability of the proposed ResASPP-Unet model. 

## 4. Discussion

### 4.1. Parameter Sensitivities of Res-ASPP-Unet, ASPP-Unet and U-Net

We examined the effects of input image bands, layer depth and number of IFMs on the ResASPP-Unet, ASPP-Unet and U-Net model performances. The results show that the overall accuracies of all three models have significantly improved by using 8-band image patches as training datasets compared to those of using 4 or 3 bands (Figure 7). Incorporating NIR band also enhanced the accuracy as it helps discriminate vegetation due to its high NIR reflectance. The combination of NIR and R, G bands produces better classification results than the combination of R, G and B bands. Although these models, like most convolution-based networks, aim to detect texture, shape, and edge features of objects by using convolution filters to facilitate image classification, we still found that spectral information is very useful in urban land cover classification task. Similar findings were also reported by [24], which found that incorporating NIR or normalized difference vegetation index (NDVI) could improve the accuracy by over 2% beyond RGB bands based on the ISPRS 2D semantic labeling color-infrared aerial imageries. Compared to the traditional RGB and NIR bands of high spatial resolution satellite imagery, the WV2 and WV3 sensors provide four extra bands including yellow, red edge and NIR2, which further facilitates the discrimination of vegetation, building roofs and water bodies. 

It is widely recognized that deep learning methods benefit from having the multiple layers. Deeper layers generate more detailed features and more abstract relationships among the features, and thus yield better performance. However, deeper layers also suggest more parameters to be learned, and the network is prone to overfitting. In this study, we found that 11 layers achieved the best accuracy, while networks with a layer number deeper than 11 did not improve the accuracies (Figure 7 and Figure 8). To an extreme situation, if the layer depth is set to 15, the size of bottom layer is reduced to 8 × 8, and there is not enough information for up-sampling. Compared to ASPP-Unet and U-Net models, we found that the ResASPP-Unet model was less sensitive to layer depth, as the information copied from shallower layers ensured that training errors did not change substantially. 

The number of IFMs determine how many different features need to be captured and learned by the network to produce the output. For simple classification task, for example, if there are only two types of classes need to be distinguished, only a few IFMs may be sufficient. For complex classification problems, a large number of IFMs are needed. In this study, we found that 64 IFMs produced higher accuracy than 80 IFMs and 48 or less IFMs. Similar to the layer depth, more IFMs indicate more parameters need to be learned and thus it needs more computational burden. Therefore, both layer depth and number of IFMs need to be carefully tuned by trial and error approaches.

### 4.2. Res-ASPP-Unet and ASPP-Unet vs. U-Net, CNN and SVM

**Figure 12:**
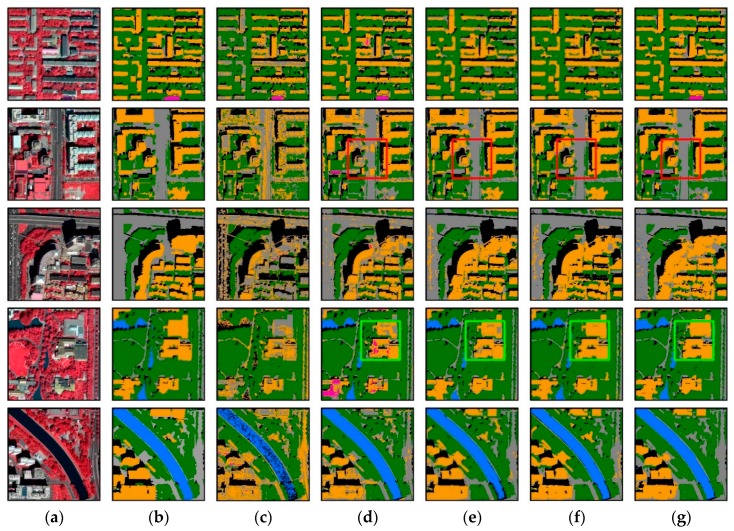
WV2 imagery classification results. (**a**) Original color-infrared images; (**b**) Ground truth; (**c**) SVM prediction; (**d**) CNN prediction; (**e**) U-Net prediction; (**f**) ASPP-Unet prediction; (**g**) ResASPP-Unet prediction.

**Figure 13:**
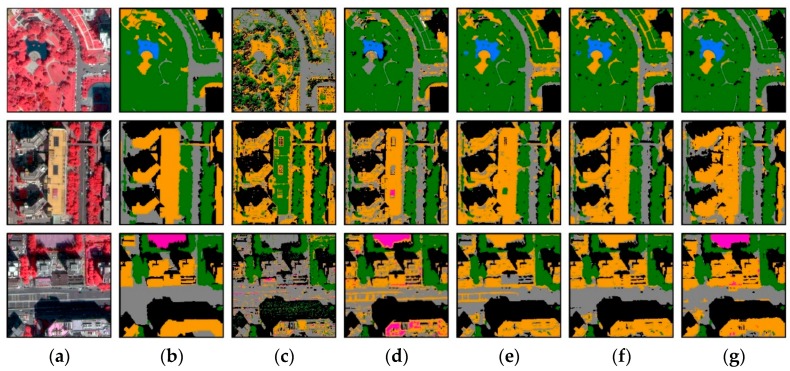
WV3 image classification results. (**a**) Original images; (**b**) Ground truth; (**c**) SVM prediction; (**d**) CNN prediction; (**e**) U-Net prediction; (**f**) ASPP-Unet prediction; (**g**) ResASPP-Unet prediction.

For both WV2 and WV3 imageries, ResASPP-Unet achieved the best classification accuracies; ASPP-Unet followed and performed better than U-Net model (Figure 7 and Figure 8, Table 2 and Table 3). Compared to the traditional machine learning SVM model, PA and UA of CNN and U-Net have significantly improved for almost all land cover types, proving the competitive power of deep learning (Table 2 and Table 3). Figure 12 and Figure 13 show the classification results over the 5 WV2 image patches and 3 WV3 image patches, respectively. It can be seen that the SVM model produces significant salt-and-pepper noise. It has difficulties in separating buildings from roads and separating water from shadow (Figure 12c and Figure 13c). Water and shadow, building and roads have similar spectral properties and thus are easily confused if only spectral information is used. Therefore, SVM, which does not incorporate convolution operations, failed in distinguishing these types. In contrast, CNN, U-Net, ASPP-Unet and ResASPP-Unet, which generate high level features via the convolution layers, substantially reduced the confusions between water and shadow, and between road and buildings. 

However, roads and buildings still tend to be confused in CNN. Dense traffic and zebra crossings on roads are misclassified as buildings (as shown as in red rectangle in Figure 12), and part of a building roof area is identified as road (green rectangle in Figure 12). In this study, the CNN was applied in a small window around the target pixel, and the target pixel’s label was then determined. Although capturing contextual information, the size of the square window was arbitrarily selected. The fixed window restricts the field-of-view of the kernel, and implies that the networks can only learn local spatial information at a single scale. In addition, the CNN increases the storage expenses and computation burden as all pixels within the squared window around each sample pixel need to be prepared and preprocessed as input data.

In contrast, the U-Net-based models can be applied more efficiently as the training and test processes are implemented in an end-to-end manner. Moreover, the test accuracies of ResASPP-Unet, ASPP-Unet or U-Net models are similar to their training accuracies, while for SVM and CNN models, test accuracies are much lower than training accuracies. This indicates that the U-Net-based models are less prone to overfitting issues owing to the dropout strategy. Compared to the U-Net model, ASPP-Unet incorporates multiple atrous convolutions in the bottom layer, thus increases the receptive fields compared to the 3 × 3 convolution in U-Net and enables capturing of the multi-scale features. With increasingly enlarged field-of-view, object contextual features at different spatial scales can be represented. In high resolution remote sensing imagery, multi-scale information is especially useful for image segmentation and classification [21,26,54]. For a given ground object, neighboring pixels usually have similar spectral information when they are located on the same object, thus using the standard filter such as 3 × 3 kernel does not necessarily capture the contextual features but causes the computation redundancy. With atrous convolution filters with variant rates, local and pixel-level features such as edges of objects as well as knowledge of the wider contextual features can be both captured. Thanks to the ASPP strategy, the confusions between building and road were further reduced. The *F*_1_ scores of both classes increase by 0.02~0.2 compared to the U-Net model. We recognized that both ASPP-Unet and U-Net models can hardly recognize the category of “others” in test phase, which can be explained by the imbalanced land cover distribution. In other words, there are very limited distribution of “others” type. ResASPP-Unet further improved the ASPP-Unet model by adding shortcut connections within each layer. The connections with identity mapping facilitate information propagation from shallower layer to deeper layers without degradation. This indicates that the networks can be more easily optimized, requiring less training samples and thus produce higher accuracies. When applying the WV2-trained models to predict WV3 land cover classes, ResASPP-Unet and ASPP-Unet also yields better performances than U-Net model (Table 3), demonstrating the improved generalization capability and robustness of the proposed models.

## 5. Conclusions

In this paper, we proposed the ASPP-Unet and ResASPP-Unet models for urban land cover classification from high spatial resolution satellite images. The proposed ASPP-Unet network combines the strength of ASPP approach and U-Net model, and allows for the extraction of multi-scale contextual information in the feature maps. In our designed model, the connections between the contracting and expanding paths of the network allow the information to be propagated through the network. ResASPP-Unet further improves ASPP-Unet architecture by incorporating residual units which allow for information propagation from shallow layers to deep layers and avoid performance degradation. Compared to U-Net, pixel-based CNN and SVM models, the ASPP-Unet achieved better accuracies (85.2%) when all models are trained and tested by using WV2 imagery over urban area. The ASPP-Unet model trained by the WV2 images also produced better classification prediction on WV3 imagery (83.2%) due to the use of multi-scale information. The ResASPP-Unet achieved the best performances benefited from the utilization of residual units, with overall accuracy of 87.1% for WV2 imagery and 84.0% for WV3 imagery, respectively.

The parameter sensitivity assessment showed that both the number of IFMs and layer depth have significant impacts on the classification accuracies. In this study, the optimal parameters are 64 IFMs and an 11-layer depth. Increasing IFMs and layer depth do not necessarily improve the model performances, but indicate greater computation complexity. Therefore, these parameters need to be carefully tuned for the classification tasks. The band selection of the input imagery also influenced the urban land cover classification accuracy. For both ASPP-Unet and U-Net models, the model trained on 8-band imagery yielded considerably higher accuracy than 4-band or 3-band imageries. Incorporating NIR band also improved the accuracy compared to RGB imagery. Although the deep learning models extensively use convolution operations to extract object features, more spectral information help to distinguish objects in the complex urban area. 

The new models proposed in this study provide an effective method for urban land cover classification. However, like any deep learning models, the models need a large number of high-quality ground truth samples as training data. Generation of these samples is labor intensive for satellite imagery over large urban area. Nevertheless, once trained, the models can be easily transferred to land cover mapping tasks in other urban areas using similar high resolution datasets. This could facilitate more efficient monitoring of urban change and provide rapid and accurate datasets for better urban planning. In the future, weak supervision networks may be developed to improve the applicability of the model for urban land cover classification tasks.

## Figures and Tables

**Table 1:** Parameters for ASPP-Unet and U-Net model training.

Fixed Parameters		Varying Input/Parameters	
Initial learning rate	0.1	Input training samples	8 bands, 4 bands (IR + RGB), RGB and CIR
Number of epoch	100	Number of IFMs	16, 32, 48, 64
Filter size	3 × 3	Layer depth	5, 7, 9, 11, 13
Pooling size	2 × 2		
Activation function	Elu		

**Table 2:** Producer’s accuracy (PA), user’s accuracy (UA), F1 score (*F*_1_), test overall accuracy (OA) and kappa coefficient (kappa) of Support Vector Machine (SVM), CNN, U-Net and ASPP-Unet models based on WorldView-2 (WV2) test image patches, and training OA of each model based on the 50 WV2 training image patches.

WV2	SVM			CNN			U-Net			ASPP-Unet			Res_ASPP_Unet		
	PA (%)	UA (%)	*F*_1_	PA (%)	UA (%)	*F*_1_	PA (%)	UA (%)	*F*_1_	PA (%)	UA (%)	*F*_1_	PA (%)	UA (%)	*F*_1_
Vegetation	91.9	91.1	0.915	92.4	93.2	0.928	91.8	93.3	0.926	91.7	94.1	0.929	91.8	94.3	0.930
Water	98.9	55.9	0.714	97.8	90.8	0.942	97.5	79.4	0.875	98.5	87.7	0.928	90.2	91.0	0.906
Road	45.6	52.4	0.488	67.7	74.4	0.709	71.8	74.5	0.731	78.0	70.1	0.738	78.5	79.9	0.792
Building	58.9	49.1	0.536	83.5	71.9	0.773	83.1	81.3	0.822	81.3	84.5	0.830	85.1	85.9	0.856
Shadow	67.7	86.7	0.796	81.1	87.8	0.843	81.8	81.7	0.817	79.0	80.3	0.796	89.0	74.9	0.814
Others	41.1	72.7	0.525	13.1	79.2	0.224	0	0	0	0	0	0	39.2	84.4	0.536
Test OA (%)		71.4			83.6			84.7			85.2			87.1	
kappa	0.606			0.774			0.788			0.793			0.820		
Training OA (%)		79.3			89.5			85.5			86.4			87.6	

**Table 3:** Producer’s accuracy (PA), user’s accuracy (UA), F1 score (*F*_1_), test overall accuracy (OA) and kappa coefficient (kappa) of SVM, CNN, U-Net and ASPP-Unet models based on WorldView-3 (WV3) test image patches.

WV3	SVM			CNN			U-Net			ASPP-Unet			Res_ASPP_Unet		
	PA (%)	UA (%)	*F*_1_	PA (%)	UA (%)	*F*_1_	PA (%)	UA (%)	*F*_1_	PA (%)	UA (%)	*F*_1_	PA (%)	UA (%)	*F*_1_
Vegetation	69.4	44.5	0.542	94.8	91.5	0.931	93.7	91.7	0.927	94.6	92.1	0.933	93.1	93.0	0.931
Water	0.12	0.01	0.001	97.0	82.7	0.892	79.1	94.3	0.860	78.4	95.5	0.861	76.8	91.4	0.835
Road	44.9	57.7	0.505	61.9	65.3	0.635	63.9	52.6	0.577	75.2	63.7	0.690	75.2	75.9	0.755
Building	29.4	32.7	0.310	67.4	60.2	0.636	69.1	81.0	0.746	69.7	83.7	0.761	74.8	80.0	0.773
Shadow	63.8	79.3	0.706	84.7	91.9	0.882	89.3	87.2	0.882	88.5	90.0	0.897	89.5	80.7	0.849
Others	0	0	0	49.6	78.9	0.609	0	0	0	0	0	0	75.8	97.0	0.851
Test OA (%)		51.3			79.2			81.0			83.2			84.0	
kappa	0.359			0.722			0.740			0.774			0.787		
